# An “Amyloid‐β Cleaner” for the Treatment of Alzheimer's Disease by Normalizing Microglial Dysfunction

**DOI:** 10.1002/advs.201901555

**Published:** 2019-11-22

**Authors:** Ruiyuan Liu, Jun Yang, Linying Liu, Zhiguo Lu, Zhuyan Shi, Weihong Ji, Jie Shen, Xin Zhang

**Affiliations:** ^1^ State Key Laboratory of Biochemical Engineering Institute of Process Engineering Chinese Academy of Sciences Beijing 100190 P. R. China; ^2^ School of Chemical Engineering University of Chinese Academy of Sciences Beijing 100049 P. R. China

**Keywords:** Alzheimer's disease, Aβ, microglia, neuroinflammation, phagocytosis

## Abstract

Alzheimer's disease (AD) is a devastating neurodegenerative disorder characterized by progressive cognitive and memory loss. The vicious circle between dysfunctional microglia and amyloid‐β (Aβ) is a crucial pathological event and accelerates the progression of AD. Herein, a zwitterionic poly(carboxybetaine) (PCB)‐based nanoparticle (MCPZFS NP) with normalizing the dysfunctional microglia and Aβ recruitment is established for the treatment of AD. Compared with the neural polyethylene glycol (PEG)‐based nanoparticles (MEPZFS NPs), the MCPZFS NPs significantly alleviate the priming of microglia by decreasing the level of proinflammatory mediators and promoting the secretion of BDNF. Most importantly, quite different from PEG, the PCB‐based NPs exhibit the behavior to recruit Aβ into microglia, which significantly enhances the Aβ phagocytosis. Moreover, the Aβ degradation is changed from the conventional lysosomal/autophagy to the proteasomal pathway in the presence of MCPZFS NPs. After the treatment with MCPZFS NPs, the Aβ burden, neuron damages, memory deficits, and neuroinflammation of APPswe/PS1dE9 mice are significantly attenuated in the brain. Therefore, the PCB‐based MCPZFS NPs have great potential to serve as an “Aβ cleaner” and provide a new insight into the therapeutic strategy for AD therapy.

## Graphical abstract

A microenvironment‐responsive zwitterionic nanoparticle (MCPZFS NP) is designed to normalize the dysfunctional microglia. The negative crosstalk between microglia and amyloid‐β (Aβ) was halted and the NPs exhibit the ability to recruit Aβ. The Aβ degradation is changed from lysosomal/autophagy to the proteasomal pathway. The NPs provide an applied horizon of zwitterionic materials and a new insight into the therapeutic strategy for Alzheimer's disease.

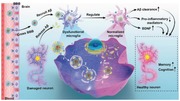

## 1 Introduction

Alzheimer's disease (AD) is the most common neurodegenerative disorder among the elderly causing progressive cognitive and memory decline. For the treatment of AD, intense focus has been directed toward inhibiting the abnormal production and aggregation of amyloid‐β (Aβ). However, thus far, numerous failures in clinical trials indicate that targeting Aβ alone is far more enough.^1^ Therefore, it is desired to consider a more efficient approach for AD treatment. As a matter of fact, the abnormal crosstalk between dysfunctional microglia and Aβ is a crucial pathological event and accelerates the AD progression. As the resident immune cells of central nervous system (CNS), microglia constantly survey the brain environment for pathogens and cellular debris.^2^ In AD brain, microglia exhibit the ability to phagocytose and degrade extracellular Aβ.^3^ However, the aberrant aggregated Aβ impair the phagocytic function of microglia and induce the generation of various proinflammatory mediators such as interleukin 1β (IL‐1β), tumor necrosis factor α (TNF‐α), interleukin 6 (IL‐6) and reactive oxygen species (ROS).^4^ In turn, these proinflammatory mediators will further facilitate the generation and aggregation of Aβ.^5^ As a result, the negative feedback loop between microglial dysfunction and Aβ accelerates AD progression.^6^ Therefore, normalizing the priming of microglia^7^ and reducing the Aβ burden^8^ are recognized as a rational and promising therapeutic strategy for AD.

For the aspect of modulate the dysfunctional microglia, fingolimod has been proved to reduce the production of inflammatory mediators in microglia.^9^ Unfortunately, fingolimod has a poor aqueous solubility, which seriously limits its application. Furthermore, the signal transducer and activator of transcription 3 (STAT3) is a key responsible gene to mediate the inflammatory cytokines secretion.^10^ The level of phospho‐STAT3 is elevated in dysfunctional microglia. Therefore, it is necessary to downregulate the expression of the SATA3 protein to play synergistic effect for inflammatory modulation. Small interfering RNA (siRNA) is considered as a great valuable therapeutic agent for downregulation of gene expression via RNA interference.^11^ In addition, it has been demonstrated that zinc oxide (ZnO) can enhance the Th2 response, which is beneficial to normalize the polarization of microglia.^12^ Inspired by the abovementioned, a codelivery of fingolimod, siSTAT3 and ZnO to normalize the dysfunctional microglia will have synergistic effects for the microglial normalization. Regrettably, the intrinsic deficiencies of these drugs, such as the short blood half‐life and poor penetrability of blood–brain barrier (BBB), restrict the therapeutic effects.^13^

Nanoparticles (NPs) have the potential to overcome these shortcomings. Neutral materials such as polyethylene glycol (PEG)‐modified NPs have been commonly used to increase the stability and blood half‐time of NPs. However, modification of PEG reduces the efficiency of cellular uptake and endosomal/lysosomal escape of NPs and results in rapid clearance in vivo, which significantly restricts the therapeutic effects.^14^ Therefore, developing a novel NP to normalize the dysfunctional microglia and reduce the Aβ burden synergistically is urgently desired. In our previous studies, the zwitterionic poly(carboxybetaine) (PCB) has been reported to enhance the cellular uptake and accelerate the endosomal/lysosomal escape of NPs, which significantly enhances the therapeutic effect.^15^ Inspired by this, a PCB‐based NP is constructed to regulate the dysfunctional microglia. Since the elevated ROS level in dysfunctional microglia, the positively charged ROS responsive polymer poly[(2‐acryloyl)ethyl(*p*‐boronic acid benzyl)dimethylammonium bromide] (PDMAEA‐BAP, PB) is chosen for siSTAT3 condensation and stimuli‐responsive release.^16^ Moreover, the mannose analog 4‐aminophenyl α‐d‐mannopyranoside (Man) is selected to guide the NPs to penetrate BBB and target to microglia.^17^ From the above, a PCB‐based NP with microglia targeting and ROS response is developed.

**Figure 1:**
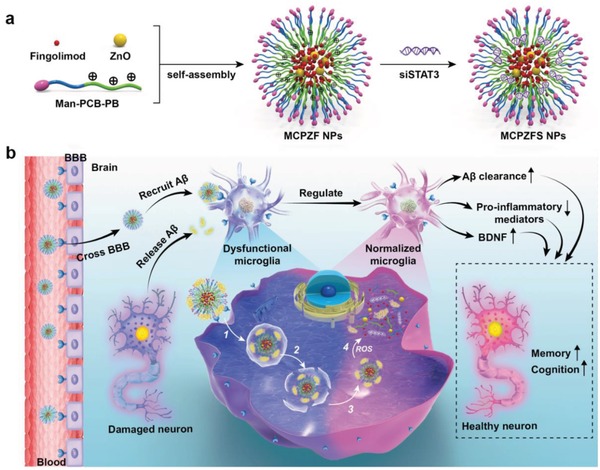
Schematic illustration of the MCPZFS NPs. a) Structural composition and preparation of the MCPZFS NPs. b) Schematic diagram of the mechanism of the MCPZFS NPs for AD therapy. The NPs crossed BBB, recruited the Aβ and were endocytosed into microglia. After the dysfunctional microglia was normalized, the secretion of proinflammatory mediators were decreased, the phagocytic ability of microglia was recovered and the production of BDNF was increased. And then the damaged neuron was repaired. 1) The MCPZFS NPs recruited Aβ and the complexes was endocytosed into microglia. 2) Then, the membrane of endosomes/lysosomes was perturbed and 3) the NPs escaped into cytoplasm. 4) The fingolimod, siSTAT3, ZnO, and Aβ were released under the ROS environment.

As shown in **Figure** **1**a, the diblock polymer Man‐PCB‐PB was synthesized and self‐assembled into NPs (**M**an‐P**C**B‐**P**B/**Z**nO/**f**ingolimod NPs, MCPZF NPs) via hydrophobic interaction to encapsulate fingolimod and ZnO in the hydrophobic region. The positively charged MCPZF NPs were condensed with siSTAT3 to form **M**an‐P**C**B‐**P**B/**Z**nO/**f**ingolimod/si**S**TAT3 NPs (MCPZFS NPs). Then the performances of the MCPZFS NPs in cellular uptake, microglial regulation and AD mice therapy were investigated. Cellular and animal studies demonstrated that the MCPZFS NPs significantly normalized the function of microglia and relieved the AD symptoms compared with the PEG‐based NPs (**M**an‐P**E**G‐**P**B/**Z**nO/**f**ingolimod NPs, MEPZFS NPs). Most excitingly, we discovered that the PCB‐based carrier could not only improve the cellular uptake and endosomal/lysosomal escape, but also recruit Aβ into microglia to enhance the Aβ clearance. Importantly, the MCPZFS NPs altered the Aβ degradation behavior through proteasomal pathway, which was quite different to that of MEPZFS NPs. Collectively, it was demonstrated that MCPZFS NP was also a promising “Aβ cleaner” for AD therapy, and provided a new horizon of zwitterionic materials for AD therapy.

The therapeutic mechanism is illustrated in Figure 1b. After crossing BBB mediated by mannose receptors, the MCPZFS NPs recruited the Aβ released from damaged neuron. And the complexes were endocytosed into microglia with the help of targeting. Subsequently, the NPs rapidly escaped from the endosomes/lysosomes by perturbation with the membranes of endosomes/lysosomes. After that, the NPs were broken up under the ROS environment after escaping into cytoplasm, and then fingolimod, siSTAT3, ZnO, and Aβ were released simultaneously. The drugs synergistically modulated the dysfunctional microglia and the Aβ was degraded. The production of BDNF was increased, the proinflammatory mediator release was reduced, and the phagocytic ability of microglia was recovered by the synergistic effect of the drugs. The damaged neuron was repaired and the AD symptom was attenuated by the drug‐based microglial regulation and PCB‐based Aβ recruitment. Therefore, the designed MCPZFS NPs would be an effectively therapeutic system and open up a new application of zwitterionic materials for AD therapy.

## 2 Results and Discussion

### 2.1 Preparation and Characterization of NPs

The synthesis route of the Man‐PCB‐PB copolymers is shown in Figure S1 (Supporting Information). The chemical structures of the monomer and polymers were characterized by ^1^H NMR spectra (Figure S2, Supporting Information). For comparison, Man‐PEG‐PB polymers were also synthesized and confirmed (Figures S3 and S4, Supporting Information). The hydrophobic ZnO NPs were synthesized via a previous method^18^ and displayed a size distribution of 3–5 nm, which were characterized by dynamic light scattering (DLS) (Figure S5, Supporting Information).

The Man‐PCB‐PB and Man‐PEG‐PB polymer could self‐assemble via hydrophobic interaction to form NPs by encapsulating fingolimod and ZnO, which were abbreviated as MCPZF NPs and MEPZF NPs, respectively. The diameter of MCPZF NPs was 84.0 nm, and MEPZF NPs had a comparable diameter of 89.4 nm (Figure S6a, Supporting Information). The zeta potential values of them were 45.0 and 25.4 mV, respectively (Figure S6b, Supporting Information). This suggested that the ability of siRNA condensation for MCPZF NPs was superior to that of MEPZF NPs. To verify these, gel‐electrophoresis assay was adopted to evaluate the complex ability of siSTAT3. MCPZF NPs completely loaded the siSTAT3 at an N/P ratio of 7. However, MEPZF NPs could only completely bind siSTAT3 at higher N/P ratio of 11 (Figure S7, Supporting Information). The results revealed that the complex capacity of MCPZF NPs was higher than that of MEPZF NPs. This might attribute to the cationic quaternary amine group of zwitterionic polymer PCB.

**Figure 2:**
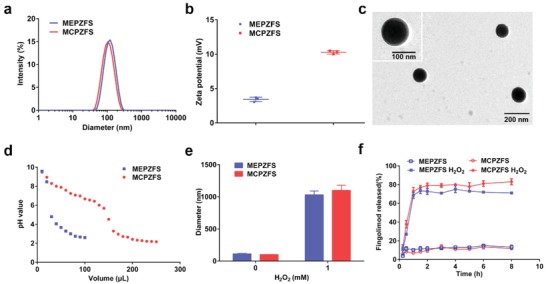
Characterization of the NPs. a) Diameter and b) zeta potential of MEPZFS NPs and MCPZFS NPs detected by DLS. c) TEM image of MCPZFS NPs; scale bar: 200 nm. The scale bar of the inset image: 100 nm. d) The buffering capacity of MEPZFS NPs and MCPZFS NPs. The buffering capacity was detected by acid–base titration in 0.01 m NaCl aqueous solution. e) The diameter change of MEPZFS NPs and MCPZFS NPs at N/P of 11 after treated with 1 × 10^−3^ m H_2_O_2_ solution for 1 h at 37 °C. f) The cumulative release of fingolimod for the NPs at 37 °C in 0.5% Tween‐80 with or without 1 × 10^−3^ m H_2_O_2_. Data are presented as the mean ± SD.

To ensure a rational comparison of the two NPs, the N/P ratio of 11 was chosen for the following experiments. The diameters of the obtained NPs were about 100 nm, which were a little increased compared with that of the cationic NPs (**Figure** **2**a). The zeta potential of MCPZFS NPs and MEPZFS NPs were decreased to 10.0 and 4.5 mV, respectively, indicating that siRNA had complexed with the cationic NPs (Figure 2b). The TEM image showed that MCPZFS NPs presented monodispersed structure (Figure 2c). MEPZFS NPs had a comparable morphology to that of MCPZFS NPs (Figure S8, Supporting Information). Next, the proton buffering capacity of the NPs were investigated by acid–based titration.^19^ Compared with MEPZFS NPs, MCPZFS NPs had a good buffering capacity over the pH range of 7.4–3.5 (Figure 2d). The pH buffering capacity of PCB indicated that the MCPZFS NPs could be protonated under the acidic condition and facilitate the endosomal/lysosomal escape.

To investigate the ROS response of the NPs, the diameter of the NPs were detected by DLS after incubating in 1 × 10^−3^ m H_2_O_2_ solution for 1 h. The diameters of both MCPZFS NPs and MEPZFS NPs were significantly increased, which was also corroborated by TEM (Figure 2e; Figure S9, Supporting Information). Next, to investigate the release kinetics of fingolimod, the NPs were incubated with or without H_2_O_2_. In the presence of H_2_O_2_, the release of fingolimod reached to ≈80% after 1 h incubation, whereas the release percentages were about 10% without H_2_O_2_ (Figure 2f). These results indicated the excellent ROS‐responsive ability of the MCPZFS NPs and MEPZFS NPs.

### 2.2 Biocompatibility, Cellular Uptake, and Endosomal/Lysosomal Escape of the NPs

The biocompatibility of NPs was crucial for the future clinical application.^20^ The cytotoxicity of MCPZFS NPs and MEPZFS NPs were evaluated by a standard methyl thiazolyl tetrazolium (MTT) assay. Murine microglial cell line BV2 was chosen as a microglia model cell. The cell viability of the two NPs were generally more than 85% at the N/P ratio from 1 to 15, suggesting these NPs were suitable for the in vivo application (Figure S10, Supporting Information).

**Figure 3:**
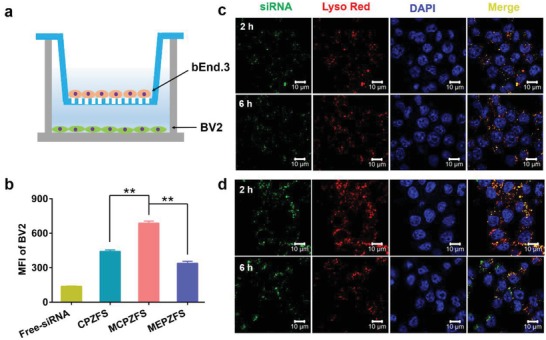
Cellular uptake and endosomal/lysosomal escape of the NPs. a) The schematic illustration of transwell model. The bEnd.3 and BV2 cells were incubated with Aβ42 oligomer for 48 h. b) The cellular uptake of NPs in BV2 cells under the transwell model of AD pathology after 2 h incubation was assayed by flow cytometry. c) The endosomal/lysosomal escape of MEPZFS NPs and d) MCPZFS NPs were assessed by CLSM after 2 and 6 h incubation, respectively. Scale bar: 10 µm. Data are presented as the mean ± SD. **P < 0.01.

As BBB was a major challenge for the drug delivery, the BBB permeability of the NPs was investigated by using a transwell model. The mouse brain endothelioma cells bEnd.3 were seeded in the upper, and BV2 cells were seeded in the bottom wells (**Figure** **3**a). When the transendothelial electrical resistance value of the bEnd.3 monolayer reached 200 Ω cm^2^, the monolayer was considered to be integrated.^21^ In order to achieve AD pathological conditions in vitro, the bEnd.3 cells and BV2 cells in the transwell model were incubated with Aβ42 oligomer for 48 h, respectively. The transwell model was considered to simulate the AD pathological condition. Then, the NPs containing Cy5‐siRNA were added to the upper culture medium, and the cellular uptake of NPs in BV2 cells was tested by flow cytometry after 2 h incubation. As shown in Figure 3b, the mean fluorescence intensity (MFI) of MCPZFS group was about 1.6 times than that of CPZFS groups without mannose targeting, suggesting that mannose could enhance the permeability of BBB and target to BV2 cells under the mimetic pathological condition of AD. Moreover, the MFI of MCPZFS group was about two times than that of MEPZFS, indicating that PCB modification had better cellular uptake ability than that of PEG. This might be attributed to the slight positive charge and brushed chemical structure of PCB,^22^ which facilitated the interactions between the NPs and cytomembrane.

After endocytosed into BV2, the endosomal/lysosomal escape of NPs was important for the subsequent drug release in the cytoplasm.^23^ BV2 cells were treated with MCPZFS NPs and MEPZFS NPs for 2 and 6 h, respectively, and the localization of NPs in cells was observed by confocal laser scanning microscope (CLSM). As shown in Figure 3c,d, the green fluorescence of both MEPZFS NPs and MCPZFS NPs were mainly colocalized with the red fluorescence of endosomes/lysosomes as yellow dot after the incubation of 2 h. Remarkably, the cell uptake of MCPZFS NPs was much more than that of MEPZFS NPs, which also demonstrated that MCPZFS NPs could enhance the cellular uptake. At 6 h, most green signal separated with the red signal of MCPZFS NPs, suggesting that the NPs had escape from endosomes/lysosomes. In contrast, MEPZFS NPs still located in the endosomes/lysosomes after 6 h of incubation. The quantified results of the overlap ratio were displayed in Figure S11 (Supporting Information). These results indicated that the modification of PCB could facilitate the endosomal/lysosomal escape by protonation and perturbation with the membranes of endosomes/lysosomes.

### 2.3 Inflammatory Inhibition of NPs

**Figure 4:**
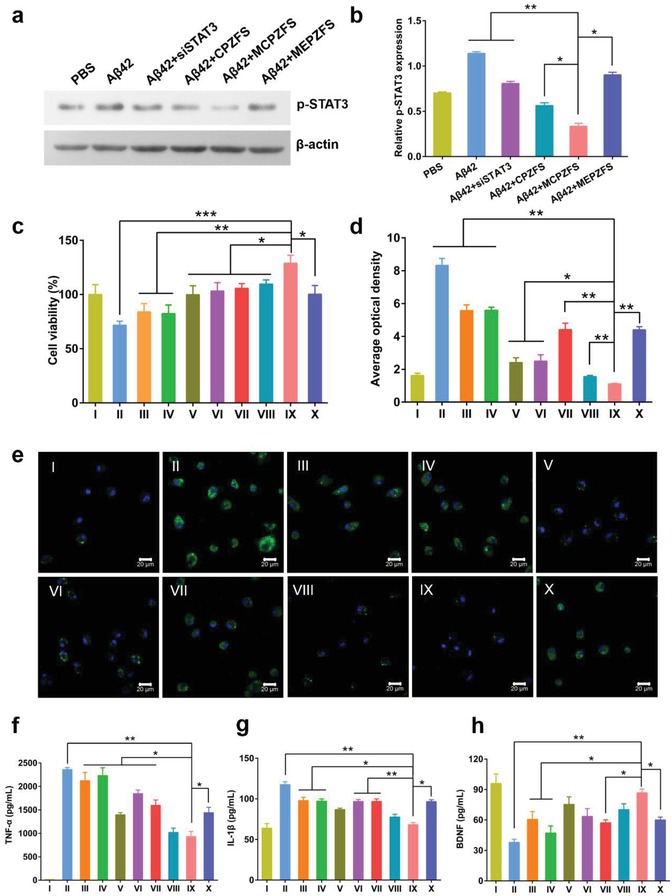
Effect of NPs on the inflammatory regulation of microglia. a) Western blot analysis of p‐STAT3 protein level under different treatment. b) Quantified results of p‐STAT3 expression as the ratio of p‐STAT3 to β‐actin from Western blot results. c) The BV2 cell viability of 24 h in each group was determined using MTT assay. d,e) The quantified and visual ROS level of primary microglia detected by CLSM under different treatments for 12 h. f) Levels of TNF‐α, g) IL‐1β, and h) BDNF in the supernatants were determined by using ELISA kits. Samples: I) PBS, II) Aβ42, III) Aβ42 + fingolimod, IV) Aβ42 + siSTAT3, V) Aβ42 + CPFS, VI) Aβ42 + CPZS, VII) Aβ42 + CPZF‐siNC, VIII) Aβ42 + CPZFS, IX) Aβ42 + MCPZFS, and X) Aβ42 + MEPZFS. Data are presented as the mean ± SD. *P < 0.05, **P < 0.01, ***P < 0.001.

Western blot was employed to detect the expression of p‐STAT3, the activated form of STAT3,^19^ to determine the silencing efficacy of the NPs. After treated with Aβ42, the expression of p‐STAT3 in BV2 cells was elevated, but the MCPZFS NPs could significantly silence the expression of p‐STAT3. However, the other groups including MEPZFS NPs only slightly decreased the level of p‐STAT3 (**Figure** **4**a,b). The result demonstrated the higher silence efficiency of PCB‐based NPs.

It was widely recognized that the aggregated Aβ42 could lead to cytotoxicity.^2^ We performed MTT assay to investigate the effect of the NPs on Aβ‐induced cytotoxicity. BV2 cells were treated with Aβ42 in the absence or the presence of NPs. As shown in Figure 4c, the cell viability was significantly reduced to 69% after incubation with Aβ42 for 24 h. Free fingolimod or siSTAT3 almost had no effect due to its poor cellular uptake. The treatment with MCPZFS NPs significantly increased the cell viability to 128%. This indicated that the PCB‐based NPs could not only remarkably reduce the Aβ‐induced cytotoxicity, but also promote the cell proliferation. However, the CPZF‐siNC or CPZS NPs could only partially enhance the cell survival. Notably, the cytotoxicity reduction of MCPZFS NPs was also superior to that of MEPZFS NPs.

Moreover, Aβ42 also induced the ROS production of microglia, which could trigger a series of damages to cellular components.^24^ Based on the performance of the MCPZFS NPs in reducing Aβ cytotoxicity, the ROS level was evaluated in microglia. The intracellular ROS content was monitored by CLSM.^25^ In the presence of Aβ42 alone, the green fluorescence was significantly increased by 5.15 times compared with the PBS group, indicating the elevated ROS level of the cells. Similar to the result of toxicity, treating the cells with MCPZFS NPs caused 7.48 times reduction to the positive control of Aβ42, which was similar to that of the cells treating with PBS (Figure 4d,e). The intracellular ROS level was also determined by flow cytometry, and the result was in accordance to that of CLSM (Figure S12, Supporting Information).

The aggregated Aβ could activate microglia and induce the secretion of proinflammatory cytokines such as TNF‐α and IL‐1β, which further induced Aβ aggregation and neuronal death.^10^ Therefore, the level of proinflammatory cytokines was evaluated by enzyme‐linked immunosorbent assay (ELISA). As displayed in Figure 4f, the supernatant level of TNF‐α was significantly increased to 2300 pg mL^−1^ for Aβ42 incubated alone. Co‐incubated with MCPZFS NPs noticeably reduced the TNF‐α production by 2.54 times compared with Aβ42 group, which was remarkably superior to that of other formulations. Notably, the TNF‐α level of MEPZFS NP treated group was 1.55 times than that of MCPZFS NPs. MCPZFS NPs also significantly suppressed the secretion of IL‐1β whereas the control groups always kept high levels (Figure 4g). In addition, the BDNF level was quantified by sandwich ELISA of different treatment. MCPZFS NPs significantly increased the BDNF level compared with the other groups, indicating the neuroprotective effect of MCPZFS NPs (Figure 4h). Hence, MCPZFS NPs could significantly inhibit the Aβ‐induced cytotoxicity, increase the production of BDNF and decrease the levels of ROS and proinflammatory cytokines, which might be attributed to the excellent capacity of cellular uptake and endosomal/lysosomal escape of PCB.

### 2.4 Microglial Phagocytosis and Degradation of Aβ

**Figure 5:**
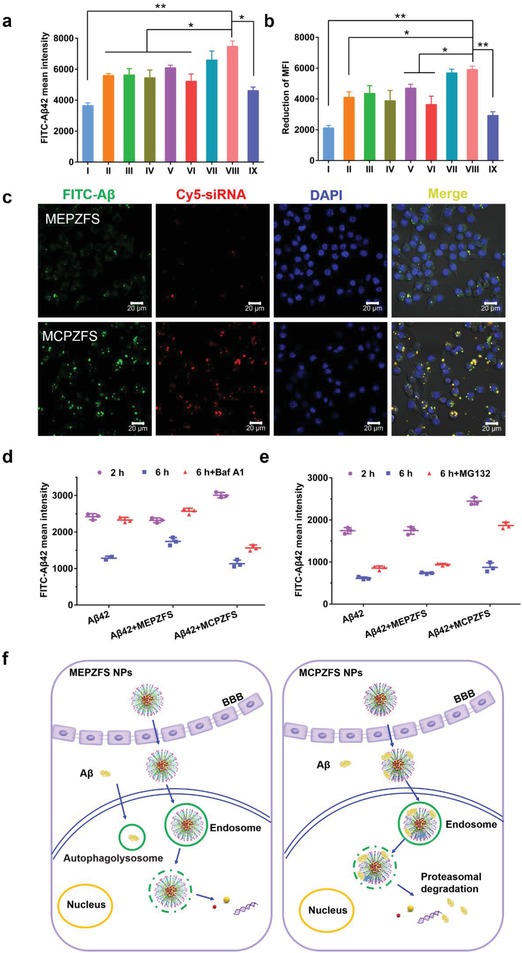
The effect of NPs on phagocytosis and degradation of Aβ by microglia. a) BV2 cells were incubated with FITC‐Aβ42 and NPs for 2 h and b) they were washed and reincubated in Aβ‐free media for another 4 h. The MFI of FITC‐Aβ42 in BV2 was determined by flow cytometry. Samples: I) Aβ42, II) Aβ42 + fingolimod, III) Aβ42 + siSTAT3, IV) Aβ42 + CPFS, V) Aβ42 + CPZS, VI) Aβ42 + CPZF‐siNC, VII) Aβ42 + CPZFS, VIII) Aβ42 + MCPZFS, and IX) Aβ42 + MEPZFS. c) BV2 cells were cocultured with Aβ42 (green, FITC‐Aβ42) and NPs (red, Cy5‐siRNA) for 1 h. The distribution of the NPs and Aβ42 were observed using confocal microscopy. Scale bar: 20 µm. d) After incubated with FITC‐Aβ42 and NPs for 2 h, the degradation of Aβ42 of BV2 for 2 to 6 h in the presence of Baf A1 or e) MG132 were detected by flow cytometry. f) Schematic diagram of the phagocytosis and degradation behavior mediated by MEPZFS NPs and MCPZFS NPs, respectively. Data are presented as the mean ± SD. *P < 0.05, **P < 0.01.

As the microglial phagocytosis of Aβ was a key mechanism for Aβ clearance, the Aβ catabolism was detected by using fluorescein isothiocyanate (FITC)‐labeled Aβ42 as a fluorescent probe. BV2 cells were incubated with Aβ42 in the presence of the NPs for 2 h and the MFI of cells were detected by flow cytometry. Co‐incubation with MCPZFS NPs resulted in a dramatic enhancement of Aβ phagocytosis compared with the Aβ group, whereas the treatment with MEPZFS NPs only slightly elevated the phagocytic uptake of Aβ (**Figure** **5**a). Next, to detect the Aβ degradation, the cells were washed after 2 h incubation and cultured in Aβ42‐free media for an additional 4 h. The treatment with MCPZFS NPs resulted in a dramatic clearance of Aβ, however, the degradation of MEPZFS group was almost comparable to that of the untreated group (Figure 5b). These results indicated that PCB could not only enhance the phagocytic uptake of Aβ, but also enhance the Aβ degradation.

The intracellular distribution of Aβ and NPs in BV2 cells was further observed by CLSM. As shown in Figure 5c, plenty of green signal of Aβ and the red signal of MCPZFS NPs were colocalized as yellow dots at 1 h, whereas only a tiny amount of green or red fluorescence could be observed for MEPZFS NPs treated cells. The quantified results of the green and red signal are displayed in Figure S13a (Supporting Information). The results indicated that MCPZFS NPs might exhibit the capacity to recruit Aβ into microglia and enhance the phagocytosis of Aβ. To confirm the assumption, whether the MCPZFS NPs could recruit Aβ was further investigated. The MCPZFS NPs were incubated with FITC‐labeled Aβ42 at room temperature for 5 min and then centrifuged. The precipitation was suspended and the fluorescence was quantified. The fluorescence intensity of MCPZFS NPs was 2.68 times than that of MEPZFS NPs, indicating that the MCPZFS NPs could effectively bind to Aβ (Figure S13b, Supporting Information). These results demonstrated that the MCPZFS NPs could recruit Aβ into microglia, which enhanced the microglial phagocytosis. This was potentially attributed to noncovalent interaction between PCB and Aβ.

Next, the Aβ degradation mechanism was examined to investigate the metabolic pathway of the NPs. In the presence of lysosomal/autophagy inhibitor bafilomycin A1 (Baf A1), the Aβ degradation of the free Aβ42 and MEPZFS group was almost completely inhibited, indicating that Aβ was degraded through the lysosomal/autophagy pathway with or without the treatment of MEPZFS NPs. However, Baf A1 could only slightly attenuate the Aβ degradation of MCPZFS group (Figure 5d). The Aβ degradation was largely inhibited by exposure to the proteasomal inhibitor MG132, indicating that MCPZFS NPs mainly mediated Aβ degradation through the proteasomal pathway (Figure 5e). These results demonstrated that MCPZFS NPs could enhance the Aβ clearance by recruiting Aβ phagocytosis and carrying it escape from lysosomes to cytoplasm rapidly before fusion with autophagosomes. Then, the Aβ was degraded by proteasomes, which was quite different from the metabolic behavior of MEPZFS NPs. Therefore, the PCB‐based NPs have great potential to act as an “Aβ cleaner” to enhance the microglial phagocytosis and degradation. The schematic diagrams of the metabolic behavior mediated by the two NPs were displayed in Figure 5f.

### 2.5 Microglial Phagocytosis, Memory Improved, and Aβ Burden in AD Mice

The BBB permeability of MCPZFS NPs labeled with FITC in APPswe/PS1dE9 transgenic mice (APP/PS1 mice) was verified via a Kodak in vivo imaging system. In comparison with the nontargeted CPZFS NPs, MCPZFS NPs could enrich in the brain more effectively after 12 h injection (Figure S14a, Supporting Information). Moreover, the brain were sectioned and stained for microglia by using Iba1 antibody. As shown in Figure S14b (Supporting Information), the majority of MCPZFS NPs signal was localized with Iba1‐labeled microglia. The results indicated that the MCPZFS NPs could effectively deliver into brain and specially target to the microglia.

**Figure 6:**
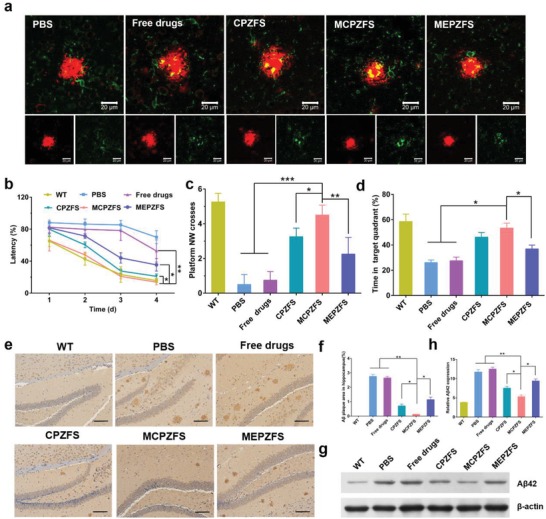
MCPZFS NPs enhanced microglial phagocytosis, reduced memory deficits and amyloid burden in APP/PS1 mice. a) Confocal images of the interaction between microglia (Iba1, green) and Aβ plaques (6E10, red) after one week treatment. b) Escape latency of the mice during MWM at 1–4 days treated with different NPs. c) Times that the mice crossed the hidden platform in the MWM on the fifth day. d) The percentage of time spent in the target quadrant during the memory test in the MWM on the fifth day. e) The immuno‐histochemical analysis images and f) quantification of 6E10‐stained Aβ deposition in the hippocampus after four weeks treatment. Scale bar: 50 µm. g) Western blot and h) quantified results of soluble Aβ in the brain homogenates of mice. Data are presented as the mean ± SD. *P < 0.05, **P < 0.01, ***P < 0.001.

Next, encouraged by the results that MCPZFS NPs could enhance the Aβ phagocytic activity of microglia in vitro, we wondered whether Aβ phagocytosis were altered in APP/PS1 mice. After one week treatment, the amyloid plaques and microglia in the cortices were detected by using immunofluorescent staining. Compared with the other groups, a significant increase of Iba1‐labeled microglia (green) was observed adjacent to the 6E10‐labeled amyloid plaques (red) with the treatment of MCPZFS NPs (**Figure** **6**a; Figure S15, Supporting Information). The result indicated that PCB‐based NPs could strikingly enhance the recruitment of amyloid plaques to microglia.

To evaluate the therapeutic effect in vivo, the NPs was intravenously administrated to 8‐month‐old APP/PS1 mice every 2 days. After four weeks treatment, Morris water maze (MWM) experiment was performed to assess the spatial learning and memory. During the 4 day training process, AD mice treated with MCPZFS NPs exhibited remarkable shorter escape latencies than that of PBS‐treated group, whereas the mice treated with free drugs, CPZFS NPs, and MEPZFS NPs only showed slightly improved (Figure 6b). Moreover, the mice treated with MCPZFS NPs also crossed the hidden platform more times and spent more swimming time in the targeted quadrant than the free drugs, CPZFS and MEPZFS NPs treated mice in the probe trail when the platform was removed (Figure 6c,d). These results demonstrated that MCPZFS NP treatment could significantly attenuate cognitive and memory impairment of AD transgenic mice.

To investigate the effect of the NPs on the Aβ burden of APP/PS1 mice, we detected Aβ deposits by using immuno‐histochemistry. The APP/PS1 mice treated with MCPZFS NPs significantly reduced the plaque area and numbers in the hippocampus and cortex compared with the PBS‐treated group and other groups (Figure 6e; Figure S16, Supporting Information). The area percentage of Aβ plaques in hippocampus were quantified by Image J software (Figure 6f). Moreover, Western blot analysis was performed to measure the soluble Aβ oligomer levels in the brain homogenates of APP/PS1 mice. Similarly, no apparent reduction of Aβ expression was detected for the free drugs treatment. The Aβ oligomer level of MCPZFS NPs treated group was significantly decreased, which was diminished by 34.7% and 48.0% compared with the CPZFS and MEPZFS NPs groups, respectively (Figure 6g,h). Moreover, the Aβ monomer levels of the treated APP/PS1 mice were measured. Similar to the result of Aβ oligomer, the MCPZFS NPs significantly reduced the level of Aβ monomer compared with other groups (Figure S17, Supporting Information). Taken together, the PCB‐modified NPs remarkably attenuated the memory impairment and amyloid burden. This was potentially attributed to the effective cellular uptake, intracellular controlled release and the enhanced Aβ phagocytosis of microglia.

### 2.6 Inflammatory Modulation and Neuron Recovery

**Figure 7:**
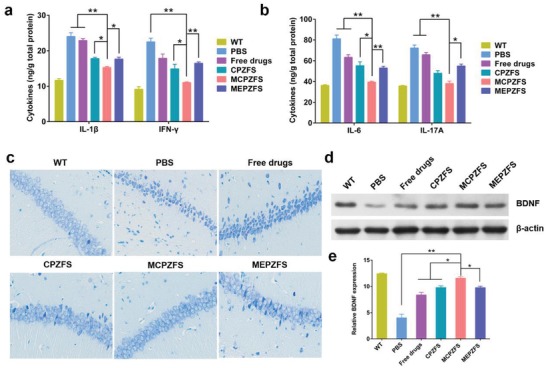
MCPZFS NPs decreased the secretion of proinflammatory cytokines and attenuated neuron damages. a) Levels of IL‐1β and IFN‐γ and b) IL‐6 and IL‐17A in the brain of wild and APP/PS1 mice after four weeks treatment. c) Nissl staining of hippocampal neurons of the wild and APP/PS1 mice after four weeks treatment. d) Western blot and e) quantified results of BDNF expression in brain after four weeks treatment. The bands were normalized to β‐actin. 200× magnification. Data are presented as the mean ± SD. *P < 0.05, **P < 0.01.

The cytokines produced by microglia were crucial for the inflammatory process of AD, the levels of several important cytokines in the cerebral homogenates were measured by using ELISA kits. Several key proinflammatory cytokines including IL‐1β, Interferon‐γ (IFN‐γ), IL‐6, and interleukin 17A (IL‐17A) were elevated in the brain of APP/PS1 mice. As expected, the levels of the proinflammatory cytokines were notably reduced by the treatment of MCPZFS NPs compared with the other groups (**Figure** **7**a,b). Then, the hippocampal neuron was analyzed by Nissl staining to assess the neuroprotective effects of the NPs. The shrinkage and loss of the hippocampal neuron were found for the PBS‐treated APP/PS1 mice. Notably, MCPZFS NP treatment significantly restored the impairment of neuronal loss and shrinkage, while free drugs, CPZFS and MEPZFS treatment did not exhibited such obvious changes (Figure 7c). In addition, Western blot analysis was performed to detect the BDNF levels, which had been proved to be strongly correlated with neuronal repair.^26^ The BDNF levels were largely reduced in the PBS‐treated APP/PS1 mice. Conspicuously, a significant increment of 2.33 times was observed for the MCPZFS NPs treated mice, which was remarkably superior to the other groups (Figure 7d,e). The result indicated that MCPZFS NP treatment could restore the levels of BDNF production of APP/PS1 mice. Additionally, the major organs of mice were collected for hematoxylin/eosin (HE) staining to assess the biosafety of the NPs. No obvious histopathological lesion was observed for all the groups (Figure S18, Supporting Information).

## 3 Conclusion

In this study, we present a microenvironment‐responsive zwitterionic nanoparticle (MCPZFS NP), which could recruit Aβ and normalize the priming of microglia by the synergistic effects of the carrier and drugs. The MCPZFS NPs could effectively permeate BBB and recruit extracellular Aβ to endocytose into microglia, which could act as an “Aβ cleaner” and cause a remarkably increase in Aβ phagocytosis. Moreover, it was discovered that MCPZFS NPs mediated Aβ degradation through proteasomal pathway due to rapid endosomal/lysosomal escape of PCB, which was quite different from the PEG‐based NPs (MEPZFS NPs) of lysosomal/autophagy pathway. Compared with the MEPZFS NPs, MCPZFS NPs significantly switch the dysfunctional microglia toward an alternative activation phenotype, which give rise to a reduction of the proinflammatory cytokines and ROS. After four weeks treatment of MCPZFS NPs, the Aβ burden, spatial learning and memory deficit, and neurologic changes of APP/PS1 mice were largely rescued. In conclusion, the study demonstrated that the MCPZFS NPs normalized the dysfunctional microglia via two synergistic approaches: 1) the fingolimod‐ and siSTAT3‐based inflammatory modulation and 2) PCB‐based Aβ recruitment of microglia. The synergistic strategy significantly hindered the crosstalk between microglia and Aβ, which attenuated the AD pathology. Therefore, the PCB‐based MCPZFS NPs provided a new horizon of zwitterionic materials for AD therapy as well as other neurodegenerative disorders.

## 4 Experimental Section

*Preparation and Characterization of the NPs*: To prepare MCPZF NPs, a 100 µL solution of Man‐PCB‐PB, fingolimod and ZnO in methanol was added dropwise into 5% glucose aqueous solution (1 mL) under strong stirring. Then the methanol was removed by dialyzing against 5% glucose aqueous solution. The MEPZF NPs were prepared with the similar method as MCPZF NPs. Next, MCPZFS NPs and MEPZFS NPs were prepared by adding STAT3 small interfering RNA (siSTAT3) to the NPs solution at various N/P ratios at room temperature for 30 min, and the siRNA binding ability of NPs was evaluated by agarose gel retardation. The diameter and zeta potential of NPs were evaluated by dynamic light scattering instrument (Malvern Zetasizer Nano ZS). The morphology of the NPs was measured by TEM (JEM‐2100 electron microscope). The drug loading of fingolimod for NPs was detected by high performance liquid chromatography (HPLC).

*In Vitro Release of Fingolimod*: 2 mL of MEPZFS NPs and MCPZFS NPs in dialysis bag (MWCO 3500) was incubated in 50 mL of 0.5% Tween‐80 with or without H_2_O_2_ (1 × 10^−3^ m) at 37 °C under horizontal shaking (150 rpm). At predetermined time intervals, 100 µL of the medium was removed and the same volume of fresh solution was supplemented. The concentration of fingolimod was detected using HPLC, which equipped with a UV detector and C18 column at 30 °C (Agilent, Japan). For the HPLC analysis, the mobile phase was a mixture of 50 × 10^−3^ m potassium dihydrogenphosphate (pH 3.0)/acetonitrile (45:55, v/v) at a flow rate of 1.0 mL min^−1^. The concentration of fingolimod was determined at the retention time of 2.7 min at 226 nm.

*Transportation across the In Vitro BBB and Cellular Uptake of NPs*: To mimetic the BBB, bEnd.3 cells were seeded in the upper chambers (pore size: 0.4 µm) of a transwell model. After the resistance value reached 200 Ω cm^2^ of bEnd.3, BV2 cells were seeded on the lower chamber. Next, the bEnd.3 cells and BV2 cells in the transwell model were incubated with 10 × 10^−6^ m Aβ42 oligomer for 48 h. NPs containing 1 µg Cy5‐siRNA were added into the upper inserts. After 2 h incubation, the mean fluorescence intensity of FAM in BV2 cells was assessed using BD Caliburflow cytometry (BD Co., USA).

*Endosomal/Lysosomal Escape of NPs*: 2 × 10^5^ BV2 cells were seeded in 35 mm Petri dishes (Cellvis) for 24 h, and then incubated with NPs including 1 µg FAM‐siRNA for 2 and 6 h at 37 °C, respectively. The cells were washed three times with PBS followed by staining with LysoTracker Red DND‐99 for 45 min at 37 °C. The cells were then washed three times with PBS and fixed with 4% paraformaldehyde for 15 min at 37 °C. Finally, the nucleus was stained with DAPI for 15 min at 37 °C. The fluorescence images were taken by CLSM (Zeiss Co., Germany).

*Aβ‐Induced Cytotoxicity Assay*: Aβ‐induced cytotoxicity was determined by the MTT assay. BV2 cells were seeded in 96‐well plates with about 1 × 10^4^ cells per well. Aβ42 (10 × 10^−6^ m) with or without the various NPs was added to the cells and the concentration of fingolimod in NPs was 200 × 10^−9^ m. After 24 h incubation, 20 µL MTT (5 mg mL^−1^) was added to the wells for another 2 h incubation. Subsequently, the medium was replaced with 100 µL DMSO each well. The absorbance was determined at 490 nm using a Tecan microplate reader (Tecan, Switzerland).

*Downregulation of p‐STAT3*: BV2 cells cultured in six‐well plates were treated with different NPs for 48 h, followed by Aβ42 (10 × 10^−6^ m) incubation for 8 h. After that, the cells were washed by PBS and then lysed for total protein extract. The level of p‐STAT3 was detected by Western blot analysis and β‐actin was used as the loading control. The western blot analysis was performed at least twice.

*Aβ‐Induced ROS and Inflammatory Cytokine Detection*: Primary microglia cells cultured in 35 mm Petri dishes (Cellvis) were treated with 10 × 10^−6^ m Aβ42 in the presence or the absence of NPs for 12 h. The concentration of fingolimod in NPs was 200 × 10^−9^ m. After that, the cells were labeled with 10 × 10^−6^ m DCFH‐DA at 37 °C for 20 min. Then, the cells were washed three times with PBS and fixed with 4% paraformaldehyde for 15 min at 37 °C. Subsequently, the nucleus was counterstained with DAPI at 37 °C for 15 min. Then, the fluorescence images were taken by CLSM (Zeiss Co., Germany). Alternatively, the cells were also collected to detect the fluorescence by BD Caliburflow cytometry (BD Co., USA). The supernatants after 12 h incubation were collected and levels of TNF‐α, IL‐1β, and BDNF were detected by using the ELISA kits according to the procedures provided by the manufacturers.

*Aβ Phagocytosis and Degradation Assay*: To evaluate the effect of the NPs on Aβ phagocytosis, BV2 cells were incubated with FITC‐Aβ (500 × 10^−9^ m) with or without NPs for 2 h. The fluorescence of BV2 cells were detected by flow cytometry analysis. For Aβ degradation, BV2 cells were washed three times after the 2 h incubation, and then cultured in Aβ‐free media with or without Baf A1 and MG132 for an additional 4 h. The fluorescence was analyzed by flow cytometry.

To further investigate the effect of NPs on Aβ phagocytosis and degradation, BV2 cells were treated with FITC‐Aβ and NPs containing Cy5‐siRNA for 1 h. Then, the cells were washed three times with PBS and fixed with 4% paraformaldehyde for 15 min at 37 °C. The nucleus was stained with DAPI at 37 °C for 15 min and then the distribution of Aβ and NPs in BV2 cells were observed by CLSM (Zeiss Co., Germany). Furthermore, FITC‐Aβ42 and NPs were incubated at 37 °C for 5 min, followed by centrifuging at 14 000 × *g* for 10 min. The fluorescence of the resuspended precipitates was quantified using microplate reader (SpectraMax M5, Molecular Devices, CA, USA) with the excitation wavelength of 488 nm and emission wavelength of 525 nm.

*In Vivo Biodistribution*: All animal experiments in this article were carried out in accordance with guidelines evaluated and approved by the ethics committee of Tsing Hua University, Beijing, China. To investigate the BBB permeability and microglia targeting ability, FITC‐labeled CPZFS and MCPZFS NPs were administrated intravenously to APP/PS1 mice. After 12 h of treatment, the mice were sacrificed and the organs (brain, heart, liver, spleen, lung, and kidney) were excised. The biodistribution of mice were detected via a Kadak in vivo imaging system. After the biodistribution studies, the brains were sectioned and stained for microglia by using Iba1 antibody.

*Drug Treatment of APP/PS1 Mice*: APP/PS1 mice (8 month old) were treated with NPs at the fingolimod dose of 0.5 mg kg^−1^ intravenously every 2 days. The mice of WT and AD control groups were treated with PBS. For studies of microglial phagocytosis of Aβ, the mice were injected for one week. For behavioral tests and therapeutic effect, mice were injected for four weeks.

*Morris Water Maze Experiments*: After four weeks of treatment, the mice were tested in a MWM (diameter, 120 cm). A 10 cm platform was fixed to 1 cm beneath the surface of water, whereas the starting position was counterbalanced. During the first 4 days, the animals were tested four times daily from four different positions around the border of the maze and they were allowed to swim for 90 s to find the platform, on which they were allowed to stay for 5 s. If the animal unable to find the platform within 90 s, it was guided and kept on the platform for 5 s. On the fifth day, the mice were tested for memory retention in a probe trial with the platform removed. Each of the mice was allowed to swim 90 s from two starting points far away from the platform.

*Immunostaining and Nissl Staining of Brain Tissue*: After the MWM test, mice were anesthetized, intracardiac perfused with PBS, and then sacrificed. The whole brains were harvested and fixed in 4% paraformaldehyde, embedded in paraffin, and sectioned at 4 µm. 6E10 antibody for amyloid plaques was performed to detect the amyloid burden. To examine the phagocytosis of Aβ, 6E10 and Iba1 antibody was employed to determine the colocalization of amyloid plaques and microglia. For Nissl staining, the brain sections were stained with cresyl violet to detect the neuronal injury in the hippocampus of mice.

*Western Blot Analysis and ELISA*: The brain tissues were dounce‐homogenized in RIPA buffer, followed by centrifuging at 14 000 × *g* for 30 min at 4 °C and the supernatant was collected. Levels of Aβ monomer, Aβ oligomer and BDNF were analyzed by Western blot. The levels of IL‐6, IL‐17A, IL‐1β, and IFN‐γ were detected by using the ELISA kits according to the procedures provided by the manufacturers.

*Statistical Analysis*: All data were expressed as mean ± SD. The significance was analyzed by the unpaired Student's *t*‐test or one‐way or two‐way ANOVA followed by Bonferroni post hoc test by using GraphPad Prism version 6.0 software.

## Conflict of Interest

The authors declare no conflict of interest.

## Supporting information

Supporting Information

## References

[1] a) D. E. Ehrnhoefer , B. K. Y. Wong , M. R. Hayden , Nat. Rev. Drug Discovery 2011, 10, 853; 22015920 10.1038/nrd3556 PMC3206090

[2] a) L. Zhong , Y. Xu , R. G. Zhuo , T. T. Wang , K. Wang , R. Z. Huang , D. X. Wang , Y. Gao , Y. F. Zhu , X. Sheng , K. Chen , N. Wang , L. Zhu , D. Can , Y. Marten , M. Shinohara , C. C. Liu , D. Du , H. Sun , L. Wen , H. Xu , G. J. Bu , X. F. Chen , Nat. Commun. 2019, 10, 1365; 30911003 10.1038/s41467-019-09118-9 PMC6433910

[3] a) A. K. Y. Fu , K. Huang , M. Y. F. Yuen , X. P. Zhou , D. S. Y. Mak , I. C. W. Chan , T. H. Cheung , B. R. Zhang , W. Fu , F. Y. Liew , N. Y. Ip , Proc. Natl. Acad. Sci. USA 2016, 113, 2705;

[4] S. W. Wang , Y. J. Wang , Y. J. Su , W. W. Zhou , S. G. Yang , R. Zhang , M. Zhao , Y. N. Li , Z. P. Zhang , D. W. Zhan , R. T. Liu , Neurotoxicology 2012, 33, 482. 22445961 10.1016/j.neuro.2012.03.003

[5] a) J. Flores , A. Noël , B. Foveau , J. Lynham , C. Lecrux , A. C. LeBlanc , Nat. Commun. 2018, 9, 3916; 30254377 10.1038/s41467-018-06449-x PMC6156230

[6] M. T. Heneka , M. J. Carson , J. E. Khoury , G. E. Landreth , F. Brosseron , D. L. Feinstein , A. H. Jacobs , T. Wyss‐Coray , J. Vitorica , R. M. Ransohoff , K. Herrup , S. A. Frautschy , B. Finsen , G. C. Brown , A. Verkhratsky , K. Yamanaka , J. Koistinaho , E. Latz , A. Halle , G. C. Petzold , T. Town , D. Morgan , M. L. Shinohara , V. H. Perry , G. Holmes , N. G. Bazan , D. J. Brooks , S. Hunot , B. Joseph , N. Deigendesch , O. Garaschuk , E. Boddeke , C. A. Dinarello , J. C. Breitner , G. M. Cole , D. T. Golenbock , M. P. Kummer , Lancet Neurol. 2015, 14, 388. 25792098 10.1016/S1474-4422(15)70016-5 PMC5909703

[7] a) D. Ofengeim , S. Mazzitelli , Y. Ito , J. P. DeWitt , L. Mifflin , C. Y. Zou , S. Das , X. Adiconis , H. B. Chen , H. Zhu , M. A. Kelliher , J. Z. Levin , J. Y. Yuan , Proc. Natl. Acad. Sci. USA 2017, 114, 8788; 28904096 10.1073/pnas.1714175114 PMC5642727

[8] a) N. Gao , Z. Du , Y. J. Guan , K. Dong , J. S. Ren , X. G. Qu , J. Am. Chem. Soc. 2019, 141, 6915; 30969760 10.1021/jacs.8b12537

[9] a) K. Fukumoto , H. Mizoguchi , H. Takeuchi , H. Horiuchi , J. Kawanokuchi , S. J. Jin , T. Mizuno , A. Suzumura , Behav. Brain Res. 2014, 268, 88; 24713151 10.1016/j.bbr.2014.03.046

[10] a) M. Eufemi , R. Cocchiola , D. Romaniello , V. Correani , L. D. Francesco , C. Fabrizi , B. Maras , M. E. Schininà , Neurochem. Int. 2015, 81, 48; 25633229 10.1016/j.neuint.2015.01.007

[11] a) M. V. Guillot‐Sestier , K. R. Doty , D. Gate , J. Rodriguez Jr. , B. P. Leung , K. Rezai‐Zadeh , T. Town , Neuron 2015, 85, 534; 25619654 10.1016/j.neuron.2014.12.068 PMC4352138

[12] a) R. Roy , S. Kumar , A. K. Verma , A. Sharma , B. P. Chaudhari , A. Tripathi , M. Das , P. D. Dwivedi , Int. Immunol. 2013, 26, 159; 24225181 10.1093/intimm/dxt053

[13] a) L. Liu , Q. Chen , L. W. Wen , C. Li , H. Qin , D. Xing . Adv. Funct. Mater. 2019, 29, 1808601;

[14] a) S. Mishra , P. Webster , M. E. Davis , Eur. J. Cell Biol. 2004, 83, 97; 15202568 10.1078/0171-9335-00363

[15] a) Y. Li , Q. Cheng , Q. Jiang , Y. Y. Huang , H. M. Liu , Y. L. Zhao , W. P. Cao , G. H. Ma , F. Y. Dai , X. J. Liang , Z. C. Liang , X. Zhang , J. Controlled Release 2014, 176, 104; 10.1016/j.jconrel.2013.12.007 24365128

[16] C. M. Qiao , J. Yang , Q. Shen , R. Y. Liu , Y. H. Li , Y. J. Shi , J. L. Chen , Y. Q. Shen , Z. B. Xiao , J. Weng , X. Zhang , Adv. Mater. 2018, 30, 1705054. 10.1002/adma.201705054 29577457

[17] a) D. Du , N. D. Chang , S. L. Sun , M. H. Li , H. Yu , M. F. Liu , X. Y. Liu , G. T. Wang , H. C. Li , X. P. Liu , S. L. Geng , Q. Wang , H. S. Peng , J. Controlled Release 2014, 182, 99; 10.1016/j.jconrel.2014.03.006 24631863

[18] Q. Shen , J. Yang , R. Y. Liu , L. Y. Liu , J. C. Zhang , S. G. Shen , X. Zhang , Mater. Horiz. 2019, 6, 810.

[19] Y. Li , Y. H. Li , W. H. Ji , Z. G. Lu , L. Y. Liu , Y. J. Shi , G. H. Ma , X. Zhang , J. Am. Chem. Soc. 2018, 140, 4164. 29486118 10.1021/jacs.8b01641

[20] Y. Chang , Y. L. Feng , Y. Cheng , R. X. Zheng , X. Q. Wu , H. Jian , D. W. Zhang , Z. H. Tang , Z. X. Wang , J. M. Hao , H. Y. Zhang , Adv. Sci. 2019, 6, 1900158. 10.1002/advs.201900158 PMC6548947 31179221

[21] Z. G. Lu , Y. Li , Y. J. Shi , Y. H. Li , Z. B. Xiao , X. Zhang , Adv. Funct. Mater. 2017, 27, 1703967.

[22] R. Zhang , Y. Li , B. B. Hu , Z. G. Lu , J. C. Zhang , X. Zhang , Adv. Mater. 2016, 28, 6345. 27168033 10.1002/adma.201600554

[23] J. Heyes , L. Palmer , K. Bremner , I. MacLachlan , J. Controlled Release 2005, 107, 276. 10.1016/j.jconrel.2005.06.014 16054724

[24] D. Felsky , T. Roostaei , K. Nho , S. L. Risacher , E. M. Bradshaw , V. Petyuk , J. A. Schneider , A. Saykin , D. A. Bennett , P. L. De Jager , Nat. Commun. 2019, 10, 409. 30679421 10.1038/s41467-018-08279-3 PMC6345810

[25] B. B. Hu , F. Y. Dai , Z. M. Fan , G. H. Ma , Q. W. Tang , X. Zhang , Adv. Mater. 2015, 27, 5499. 26270904 10.1002/adma.201502227

[26] J. A. M. Coull , S. Beggs , D. Boudreau , D. Boivin , M. Tsuda , K. Inoue , C. Gravel , M. W. Salter , Y. De Koninck , Nature 2005, 438, 1017. 16355225 10.1038/nature04223

