# Supplementary material for: An “Amyloid‐β Cleaner” for the Treatment of Alzheimer's Disease by Normalizing Microglial Dysfunction
Source: Adv Sci (Weinh). 2019 Nov 22;7(2):1901555. doi: 10.1002/advs.201901555 (PMC6974948; doi:10.1002/advs.201901555)
Supplement: Supplementary file 1 — Supporting Information [file ADVS-7-1901555-s001.pdf]

Copyright WILEY-VCH Verlag GmbH & Co. KGaA, 69469 Weinheim, Germany, 2019.

ADVANCED SCIENCE
Open Access

Supporting Information

for *Adv. Sci.,* DOI: 10.1002/advs.201901555

An “Amyloid-β Cleaner” for the Treatment of Alzheimer’s Disease by Normalizing Microglial Dysfunction

*Ruiyuan Liu, Jun Yang, Linying Liu, Zhiguo Lu, Zhuyan Shi, Weihong Ji, Jie Shen, and Xin Zhang**

Supporting Information

An 'Amyloid-β Cleaner' for the Treatment of Alzheimer's Disease by Normalizing Microglial Dysfunction

*Ruiyuan Liu†, Jun Yang†, Linying Liu, Zhiguo Lu, Zhuyan Shi, Weihong Ji, Jie Shen, and Xin Zhang**

1. Experimental Section

1.1. Materials.

2-(N,N'-dimethylamino) ethyl methacrylate (DMAEMA, 98%), β-propiolactone (98%), 2-(dimethylamino)-ethyl acrylate (DMAEA, 98%), 4-cyano-4-(dodecylsulfanylthiocarbonyl) sulfanylpentanoic acid (CTA), 4-(bromomethyl)phenylboronic acid (98%) 4-aminophenyl α-D-mannopyranoside (Man) (98%), fingolimod and 1,1,1,3,3,3-hexafluoro-2-propanol (HFIP) were obtained from J&K Scientific Ltd., Co (Shanghai, China). 2,2'-Dicyano-2,2'-azopropane (AIBN) was purchased from Aladdin industrial Corporation. NH_2-PEG-COOH was provided by Yarebio Ltd. Co (Shanghai, China). Aβ42 was obtained from GL Biochem (Shanghai). SiRNA against STAT3 (sense strand, 5'-GGACGACUUUGAUUUCAACTT-3'; antisense strand, 5'-GUUGAAAUCAAAGUCGUCCTT-3') were synthesized by GenePharma company (Suzhou, China). 4,6-Diamidino-2-phenylindole dihydrochloride (DAPI), 3-(4,5-dimethylthiazol-2-yl)-2,5- diphenyltetrazolium bromide (MTT), BCA protein assay kit were from Solarbio Science & Technology Ltd, Co (Beijing, China). The ROS kit were obtained from Beyotime Co. (Jiangsu, China). Lysotracker Red DND-99 and all ELISA Kits were purchased from Invitrogen (Carlsbad, CA, USA). Phospho-STAT3 (Tyr705) (p-STAT3) antibody was purchased from Affinity (Jiangsu, China). All the reagents were used as received without further purification. High-purity water (Milli-Q Integral) with a conductivity of 18 MΩ cm^{-1} was used for the preparation of all aqueous solutions.

1.2. Supplementary Methods

Synthesis of poly(carboxybetaine) (PCB): CB monomer were performed according to our previous report.[1] Briefly, to obtain CB monomer, DMAEMA (0.79 g, 5 mmol), β-propiolactone (0.43g, 6 mmol) and 5 mL of anhydrous CH_2Cl_2 were mixed and added into a dry and clean glass tube, followed by degassing three freeze-pump-thaw cycles and recharged with nitrogen. After that, the reaction mixture was stirred at low temperature for 12 h. The precipitate was washed with diethyl ether and then dried under reduced pressure to obtain CB product. The ^1H NMR (Bruker 600 MHz, D_2O, δ ppm) was carried out to characterize the obtained product.

PCB was synthesized by the reversible addition-fragmentation chain transfer polymerization (RAFT) polymerization. Briefly, CB (1 g, 4.35 mmol), 4-cyano-4-(dodecylsulfanylthiocarbonyl) sulfanylpentanoic acid (CTA) (176 mg, 0.436 mmol) and AIBN (29.35 mg) were dissolved in methanol and then the mixture was transferred to a dried schlenk flask. The Schlenk flask was degassed by three freeze-pump-thaw cycles and recharged with nitrogen and the reaction mixture was stirred at 65 ºC for 24 h. The resulting liquid was dialyzed in a cellu SepH1-membrane (MWCO3500) against ethanol and deionized water sequentially to remove the unreacted monomers and impurities. After 48 h dialysis, the liquid was freeze-dried to get the final product PCB. The ^1H NMR (Bruker 600 MHz, DMSO-d_6, δ ppm) was carried out to characterize the obtained product.

Synthesis of PCB-PB polymers: PCB-PDMAEA was synthesized by the RAFT polymerization. The PCB (58 mg, 0.025 mmol), DMAEA (357.5 mg, 2.5 mmol) and AIBN (1.68 mg) were dissolved in N,N-Dimethylformamide (DMF) and then added into a clean and dry schlenk flask for RAFT polymerization. The product was dialyzed in a cellu SepH1-membrane (MWCO3500) against DMF and deionized water sequentially to remove the unreacted PDMAEA and impurities. The final product PCB-PDMAEA was obtained after

lyophilized. The ^{1}H NMR (Bruker 600 MHz, DMSO-d_6, δ ppm) was carried out to characterize the obtained product.

Next, PCB-PDMAEA (296.5 mg, 1.58 mmol tertiary amines) and 4-(bromomethyl) phenylboronic acid (509 mg, 2.36 mmol) were dissolved in 5 mL DMF and stirred at room temperature for 12 h. The resulting liquid was dialyzed in a cellu SepH1-membrane (MWCO3500) against DMF and deionized water sequentially and then freeze-dried to obtain the final product PCB-PB. The ^{1}H NMR (Bruker 600 MHz, CD_4O, δ ppm) was carried out to characterize the obtained product.

Synthesis of Man-PCB-PB: PCB-PB (500 mg, 0.1 mmol) was dissolved in anhydrous methanol and activated by EDC/NHS for 30 min. Then 4-aminophenyl α-D-mannopyranoside (80.14 mg, 0.3 mmol) was added into the mixture dropwise and stirred for 24 h at room temperature. After the reaction, the final product was dialyzed and lyophilized to obtain Man-PCB-PB. The ^{1}H NMR (Bruker 600 MHz, CD_4O, δ ppm) was carried out to characterize the obtained product.

Synthesis of PEG-PB polymers: CTA (40.37 mg, 0.1 mmol) was dissolved in anhydrous DMF, and the carboxyl was activated by EDC/NHS for 0.5 h. Then NH_2-PEG2000-COOH was added into the mixture dropwise and stirred at room temperature for another 10 h. The resulting solution was dialyzed against DMF and deionized water sequentially, and then lyophilized. The ^{1}H NMR (Bruker 600 MHz, DMSO-d_6, δ ppm) was carried out to characterize the obtained product.

After NH_2-PEG2000-COOH was modified by CTA, the comparison group Man-PEG-PB polymer was synthesized with the similar route of PCB-PB. The ^{1}H NMR (Bruker 600 MHz, CD_4O, δ ppm) was carried out to characterize the obtained product.

Synthesis of ZnO nanoparticles: ZnO NPs were prepared using the reported method. Briefly, zinc acetate (403.6 mg, 2.2 mmol) and NaOH (100 mg, 2.5 mmol) were dissolved in refluxing ethanol, respectively. After cooled down, the NaOH solution was injected into the

zinc acetate solution rapidly and stirred for 1 h in ice bath. Then the product was precipitated by using n-hexane to obtain ZnO NPs.

ROS-responsive ability: The NPs were incubated in 1 mM H_2O_2 solution at 37 ºC for 1 h. After that, the diameters were measured by DLS instrument.

Detection of buffering capacity: The buffering capacity of NPs was determined by acid-base titration. Briefly, the PEG or PCB modified NPs solution were adjusted to pH 10 with 1 M NaOH. The solution was titrated by the stepwise addition of 0.1 M HCl to obtain the titration profile.

Preparation of oligomeric Aβ42: Aβ42 was dissolved in HFIP and dried under a gentle stream of nitrogen. The Aβ42 peptide film was resuspended in dimethylsulfoxide (DMSO) and diluted in PBS to a final concentration of 100 µM. Then the solution was incubated at 4 ºC for 24 h and centrifuged at 14,000×g for 10 min. The supernatant was stored at -20 ºC.

Cell culture: BV2 cells were obtained from China Academy of Medical Sciences tumor cell bank (Beijing, China). The bEnd.3 cells were obtained from Shanghai Enzyme Biological Technology Co., Ltd (Shanghai, China). Primary microglia were isolated from the brains of neonatal 1 day old mice according to procedures described previously.[2] All of the cells were cultured in DMEM (hyclone, Logan, UT) containing 10% fetal bovine serum (FBS) and 1% antibiotics (penicillin/streptomycin) at 37 ºC under 5% CO_2 environment.

Cytotoxicity of NPs: BV2 cells were cultured into 96-well plates at a density of 1×10^4 cells per well. The cells were treated with NPs and siSTAT3 at different ratios of N/P for 24 h, 20 µL MTT solution (5 mg/mL) was added to each well and incubated for additional 2-4 h. The medium was aspirated gently from each well and 100 µL of DMSO was added. The absorbance at 490 nm was measured using a Microplate reader (Tecan, Switzerland).

2. Supplementary Figures

Table S1. The abbreviation of formulations prepared in the experiments

Full name	Abbreviation
Free fingolimod & siSTAT3	Free drugs
PCB-PB/fingolimod/siSTAT3	CPFS
PCB-PB/ZnO/siSTAT3	CPZS
PCB-PB/ZnO/fingolimod/siNonsense	CPZF-siNC
PCB-PB/ZnO/fingolimod/siSTAT3	CPZFS
Man-PCB-PB/ZnO/fingolimod/siSTAT3	MCPZFS
Man-PEG-PB/ZnO/fingolimod/siSTAT3	MEPZFS

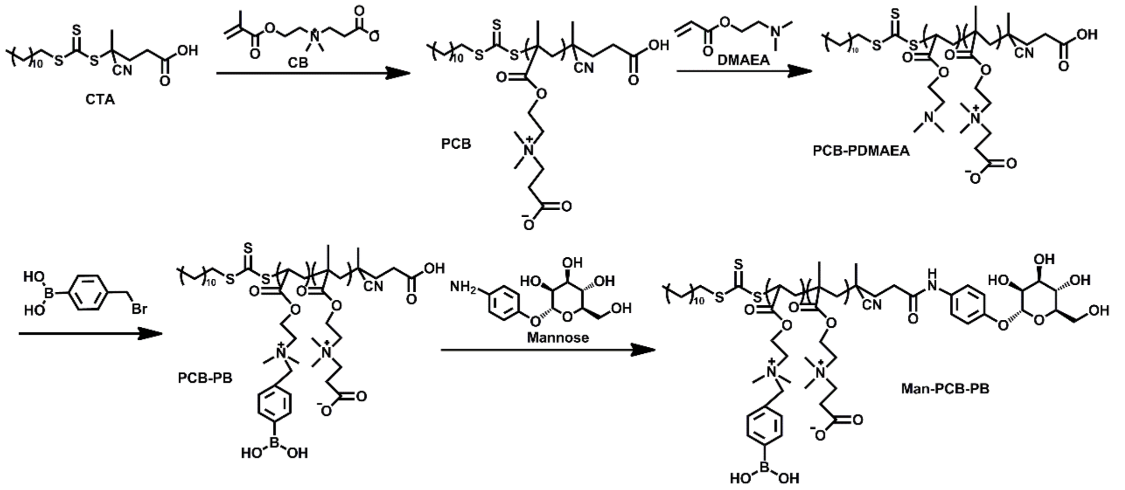

Figure S1. The synthetic routes of Man-PCB-PB.

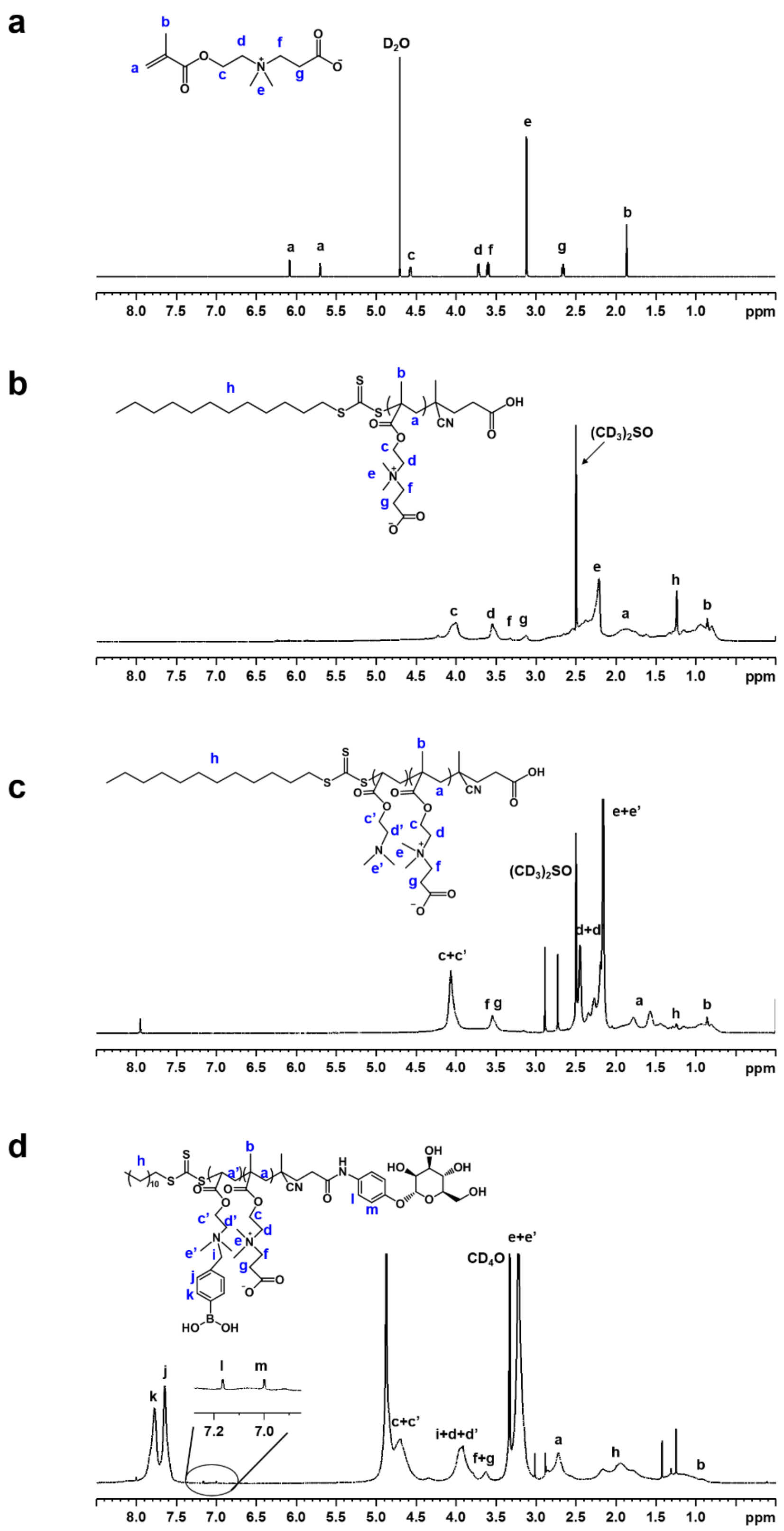

Figure S2. a) ^{1}H NMR spectra of the CB monomer, b) PCB, c) PCB-PDMAEA, d) Man-PCB-PB.

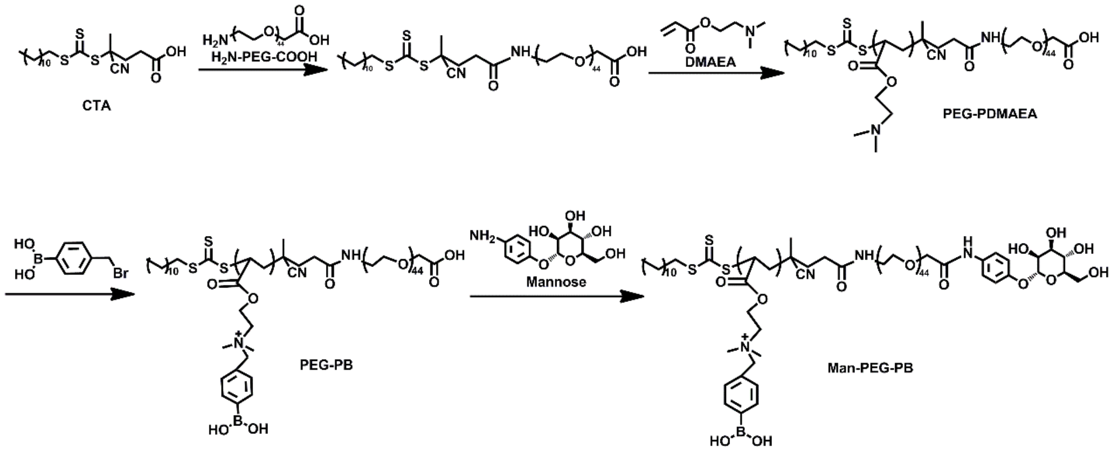

Figure S3. The synthetic routes of Man-PEG-PB.

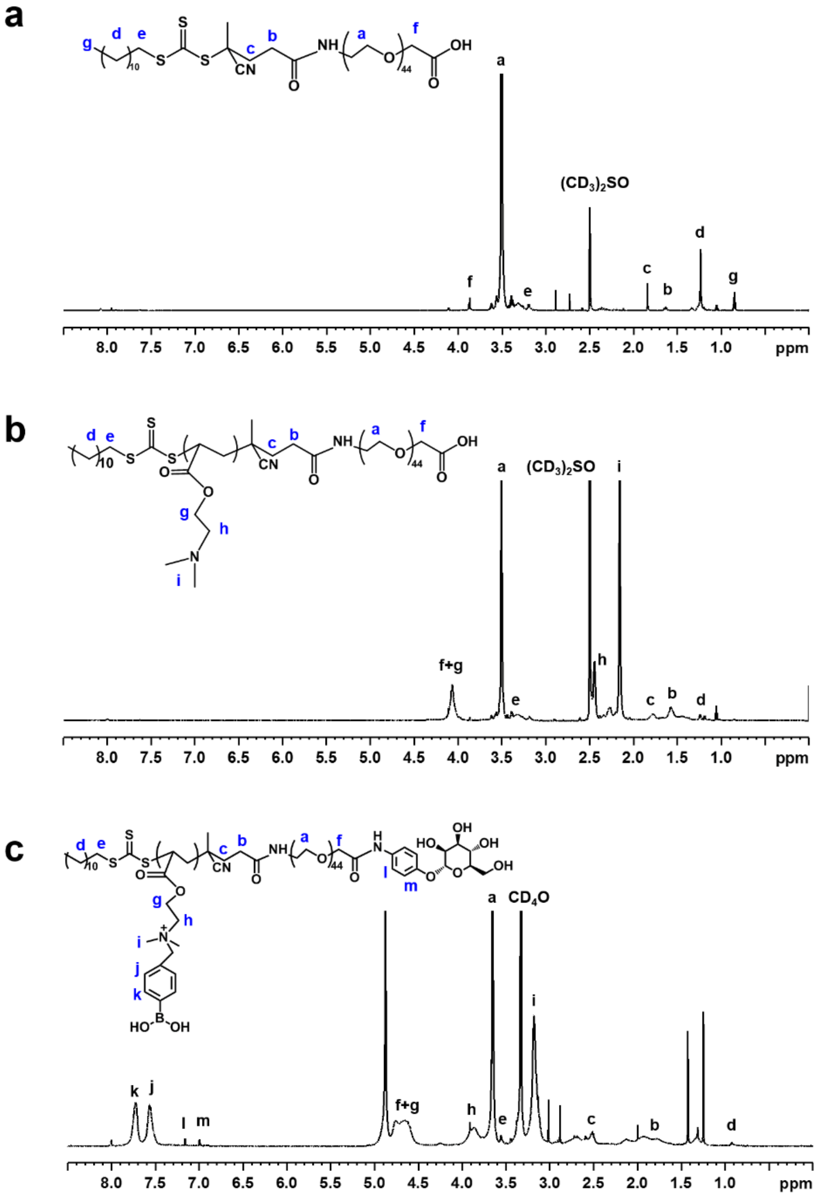

Figure S4. a) ^{1}H NMR spectra of the PEG-CTA, b) PEG-PDMAEA and c) Man-PEG-PB.

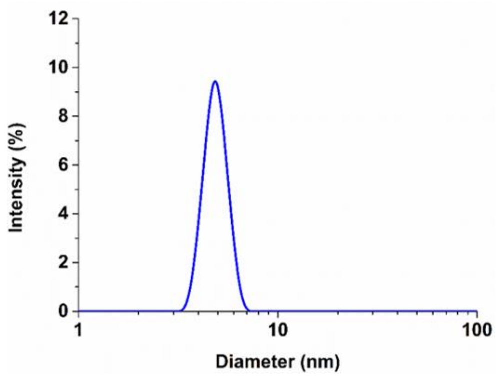

Figure S5. The size distribution of ZnO NPs measured by DLS.

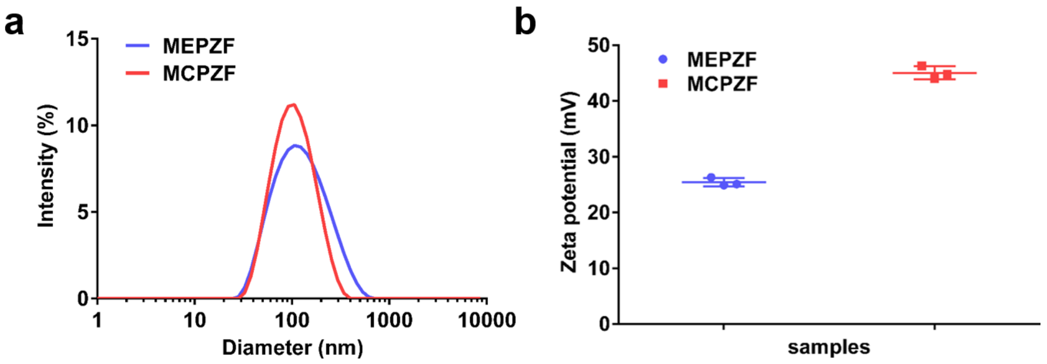

Figure S6. a) The diameter and b) zeta potential of the MEPZF and MCPZF NPs by DLS.

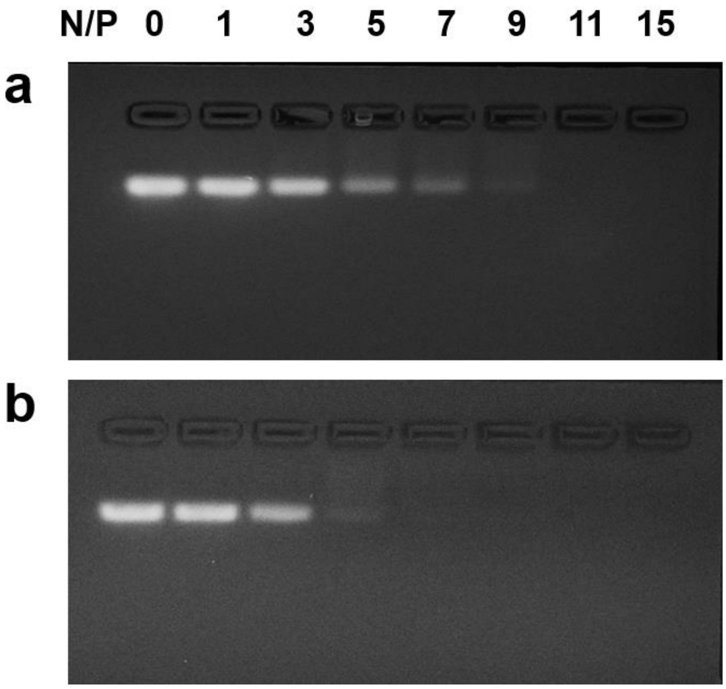

Figure S7. a) The gel retardation assay of siRNA at various N/P ratios of the MEPZFS NPs and b) MCPZFS NPs.

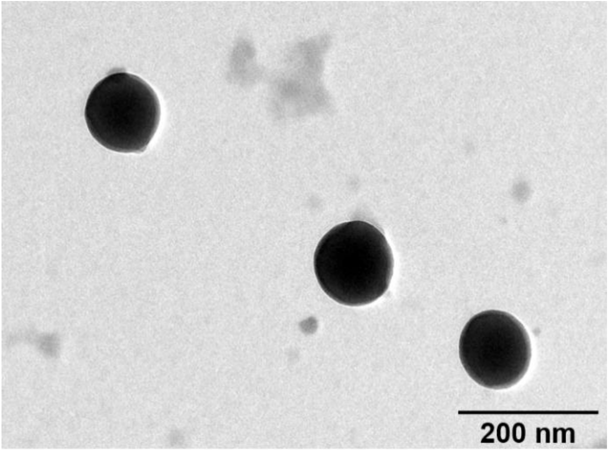

Figure S8. TEM image of MEPZFS NPs, scale bar: 200 nm.

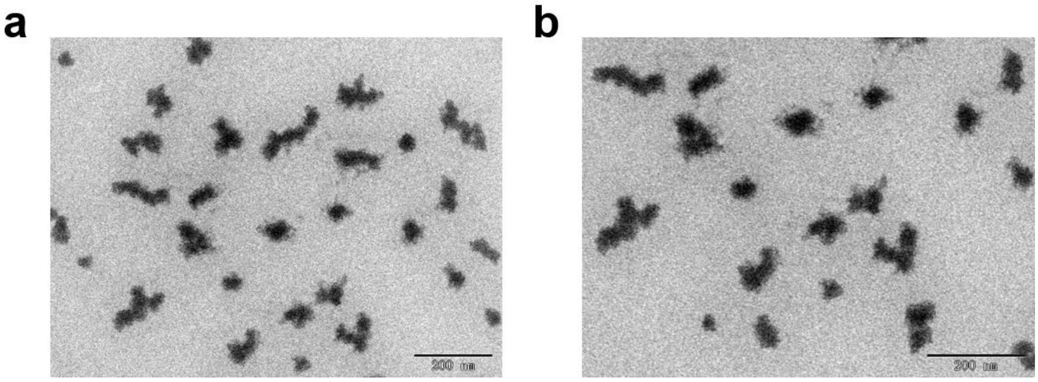

Figure S9. a) TEM images of MEPZFS and b) MCPZFS incubated in 1 mM H_2O_2 at 37 ºC for 1 h. Scale bar: 200 nm.

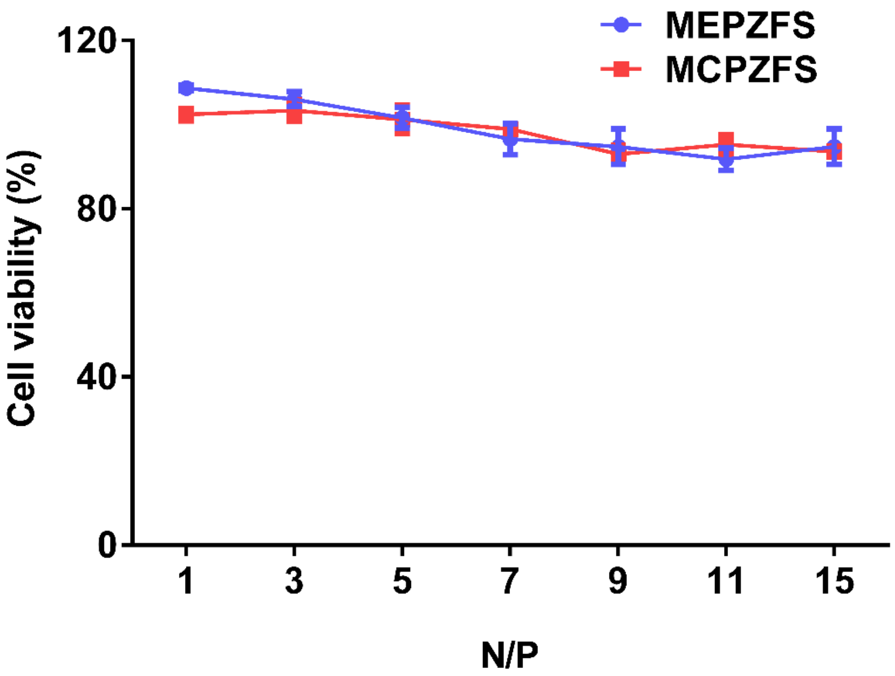

Figure S10. Cell viability of NPs after 24 h treatment with different N/P ratios in BV2 cells. Data are presented as the mean ± SD.

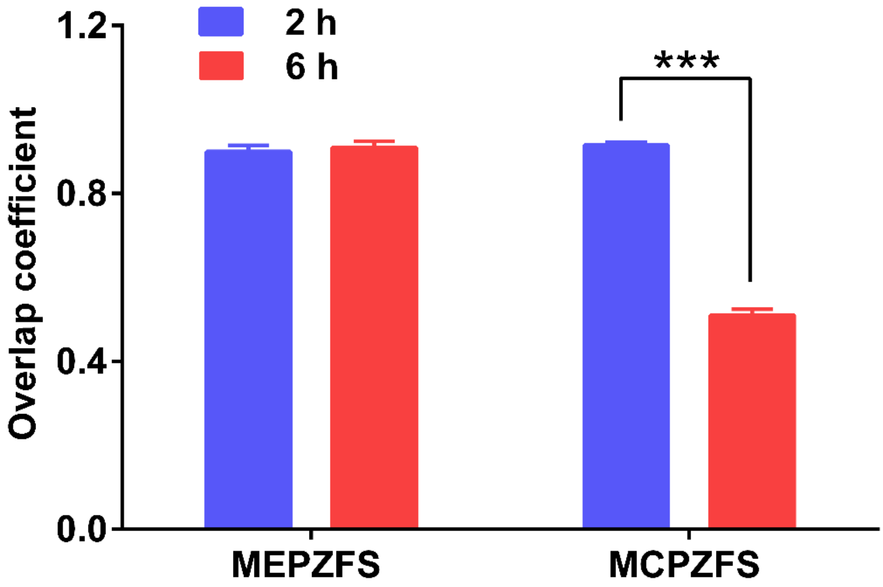

Figure S11. Overlap coefficient of FAM-siRNA and LysoTracker Red of Figure 3c,d was quantified by Image J software. Data are presented as the mean ± SD. ****P* < 0.001.

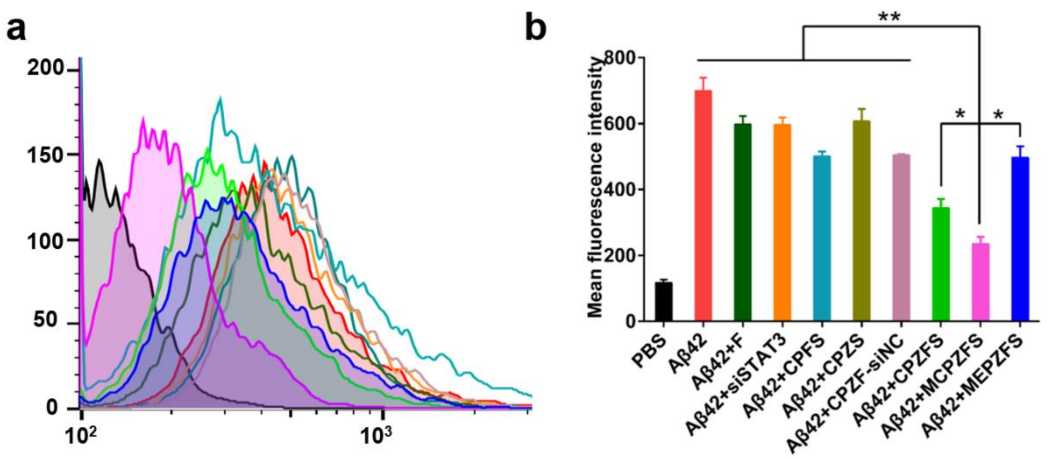

Figure S12. a) The intracellular ROS level of BV2 cells detected by flow cytometry of different treatments for 12 h and b) quantified results. Data are presented as the mean ± SD. *P < 0.05, **P < 0.01.

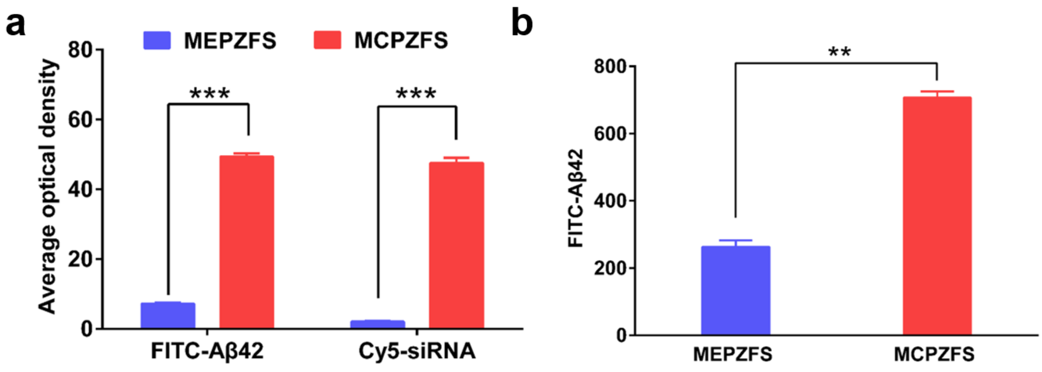

Figure S13. a) The average optical density of FITC-Aβ42 and Cy5-siRNA of the cells with different treatment. b) Detection of the absorption of NPs and Aβ. Data are presented as the mean ± SD. $^{**}P < 0.01$, $^{***}P < 0.001$.

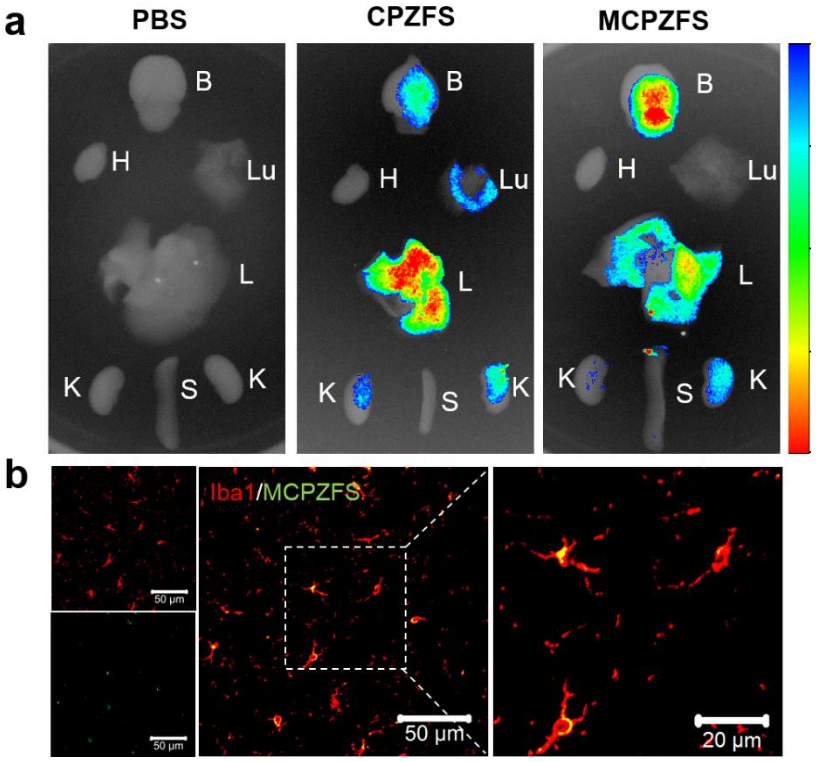

Figure S14. a) *Ex-vivo* biodistribution of NPs in PBS, CPZFS and MCPZFS NPs treated APP/PS1 mice after 12 h *via* intravenous injection. ((heart (H), lung (Lu), liver (L), kidney (K), spleen (S) and brain (B)). b) Immunofluorescence staining of MCPZFS NPs treated APP/PS1 mice brain: microglia (red) and FITC-labeled MCPZFS NPs (green).

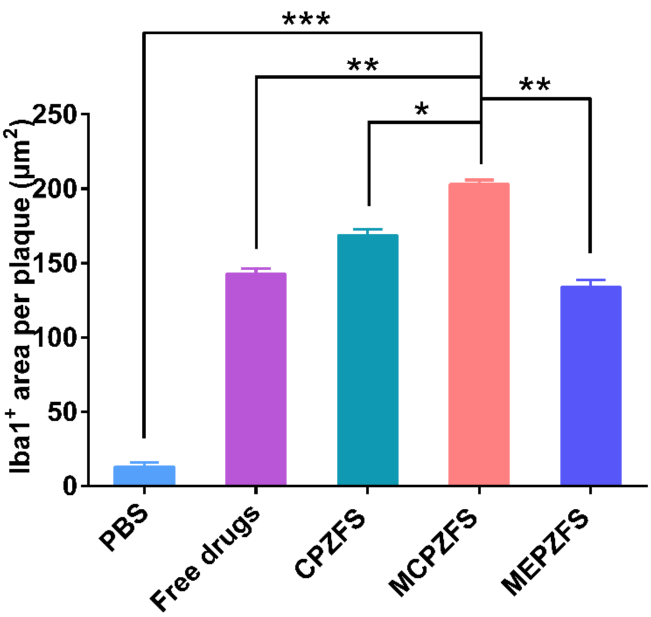

Figure S15. Area of Iba1[+] labeling per plaque was measured by Image J software. Data are presented as the mean ± SD. **P* < 0.05, ***P* < 0.01, ****P* <0.001.

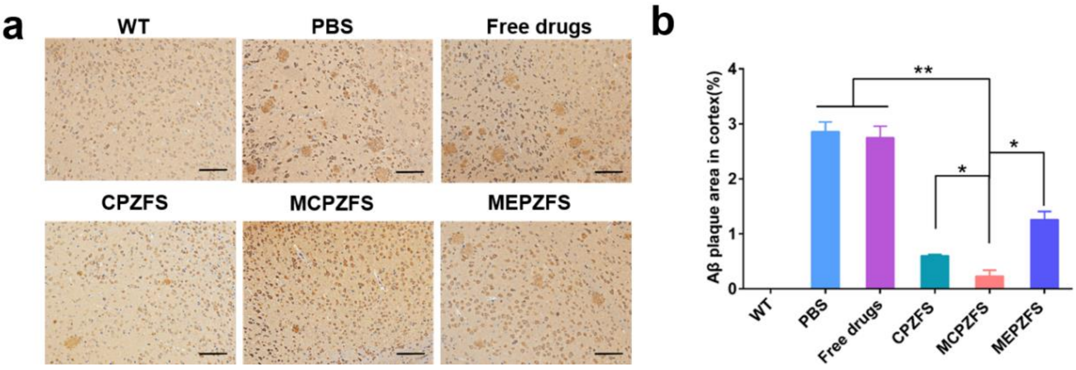

Figure S16. a) The immunohistochemical analysis of Aβ deposition and b) quantification in cortex using 6E10 antibody. Scale bar: 50 μm. Data are presented as the mean ± SD. *$P < 0.05$, **$P < 0.01$.

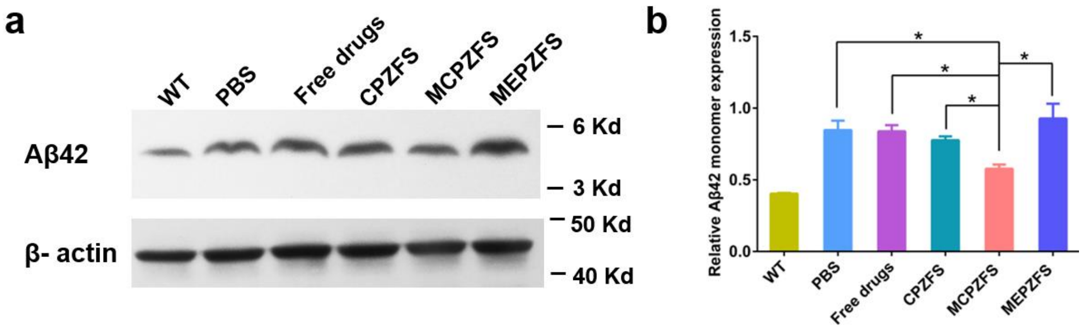

Figure S17. a) Western blot and b) quantified results of Aβ monomers in the brain homogenates of APP/PS1 mice. The bands were normalized to β-actin. Data are presented as the mean ± SD. *$P < 0.05$.

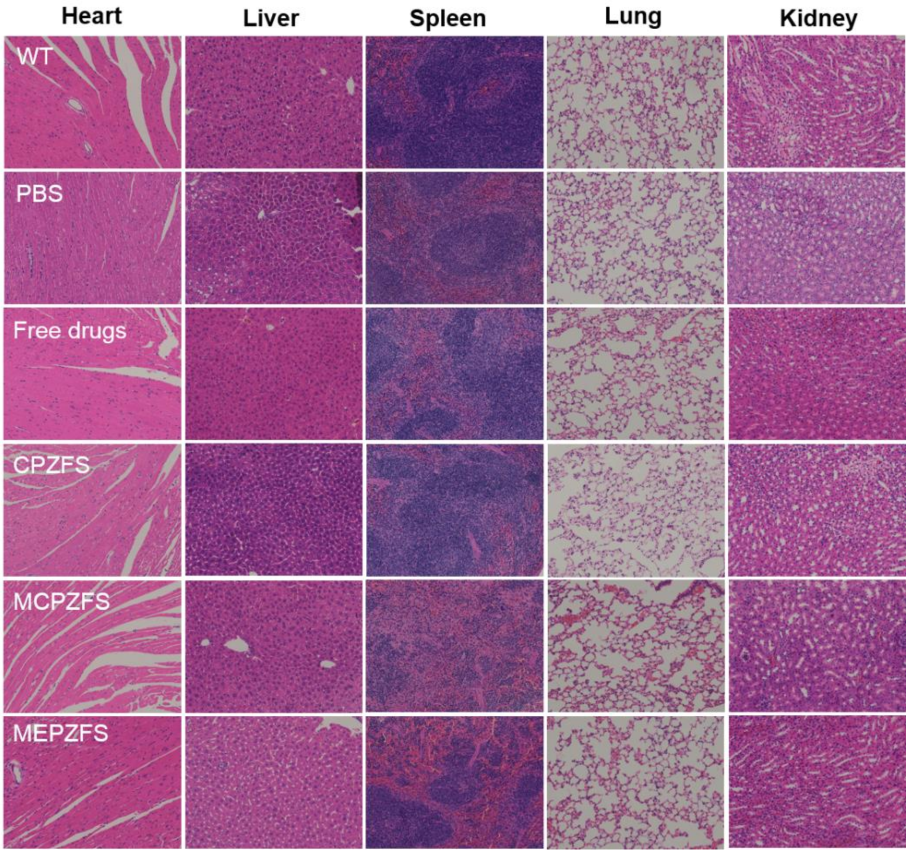

Figure S18. Representative HE staining images of major organs from the treated mice. Magnification: 200×.

References

[1] Y. Li, Q. Cheng, Q. Jiang, Y. Y. Huang, H. M. Liu, Y. L. Zhao, W. P. Cao, G. H. Ma, F. Y. Dai, X. J. Liang, Z. C. Liang, X. Zhang, *J. Controlled Release* **2014**, *176*, 104.

[2] C. Y. D. Lee, A. Daggett, X. F. Gu, L. L. Jiang, , P. Langfelder, X. G. Li, N. Wang, Y. J. Zhao, C. S. Park, Y. Cooper, I. Ferando,; I. Mody, C. Coppola, H. X. Xu, W. Yang, *Neuron* **2018**, *97*, 1032.
